# Europium Doping Impact on the Properties of MBE Grown Bi_2_Te_3_ Thin Film

**DOI:** 10.3390/ma13143111

**Published:** 2020-07-13

**Authors:** Katarzyna Balin, Marcin Wojtyniak, Mateusz Weis, Maciej Zubko, Bartosz Wilk, Ruizhe Gu, Pascal Ruello, Jacek Szade

**Affiliations:** 1 Institute of Physics and Silesian Center for Education and Interdisciplinary Research, University of Silesia, 75 Pułku Piechoty 1A, 41–500 Chorzów, Poland; marcin.wojtyniak@us.edu.pl (M.W.); mateusz.weis@ensta-paris.fr (M.W.); bartosz.wilk@smcebi.edu.pl (B.W.); jacek.szade@us.edu.pl (J.S.); 2 Institut des Molécules et Matériaux du Mans, UMR CNRS 6283, Université le Mans, 72085 Le Mans, France; ruizhe.gu.etu@univ-lemans.fr (R.G.); pascal.ruello@univ-lemans.fr (P.R.); 3 Institute of Materials Engineering, University of Silesia, 75 Pułku Piechoty 1A, 41–500 Chorzów, Poland; maciej.zubko@us.edu.pl

**Keywords:** topological insulators, Bi_2_Te_3_ thin films, doping, electronic structure, europium valence, electron-phonon interaction

## Abstract

The impact of europium doping on the electronic and structural properties of the topological insulator Bi_2_Te_3_ is studied in this paper. The crystallographic structure studied by electron diffraction and transmission microscopy confirms that grown by Molecular Beam Epitaxy (MBE) system film with the Eu content of about 3% has a trigonal structure with relatively large monocrystalline grains. The X-ray photoemission spectroscopy indicates that europium in Bi_2_Te_3_ matrix remains divalent and substitutes bismuth in a Bi_2_Te_3_ matrix. An exceptional ratio of the photoemission 4d multiplet components in Eu doped film was observed. However, some spatial inhomogeneity at the nanometer scale is revealed. Firstly, local conductivity measurements indicate that the surface conductivity is inhomogeneous and is correlated with a topographic image revealing possible coexistence of conducting surface states with insulating regions. Secondly, Time of Flight Secondary Ion Mass Spectrometry (TOF-SIMS) depth-profiling also shows partial chemical segregation. Such in-depth inhomogeneity has an impact on the lattice dynamics (phonon lifetime) evaluated by femtosecond spectroscopy. This unprecedented set of experimental investigations provides important insights for optimizing the process of growth of high-quality Eu-doped thin films of a Bi_2_Te_3_ topological insulator. Understanding such complex behaviors at the nanoscale level is a necessary step before considering topological insulator thin films as a component of innovative devices.

## 1. Introduction

The efficiency, stability, and lifetime of any device is strongly dependent on the stability of compounds and alloys that a given device is made of. The attempts to understand the particular state of matter are crucial points of understanding its implications for application in advanced electronic devices. This gain in importance when exotic states of matter are considered. Certain exotic behavior, namely the insulating character of the bulk and metallic character of the surface, was observed in a large group of materials, which were later classified as topological insulators [1,2]. Among classified topological insulators the Bi_2_Te_3_ compound, considered as good material for thermoelectric [3,4] and optoelectronic [5,6] applications, was selected as the subject of this study. Topological insulators (TIs) are a novel type of quantum materials of which the bulk part is a band insulator whereas the surface always exhibits the presence of electronic states carrying electric current [2,7]. This phase shows a clear bulk energy gap separation like any ordinary insulator or semiconductor, but the difference is in the presence of the gapless edge states (2D) or surface states (3D) which are protected by time-reversal symmetry. The unusual nature of the TI surface states resulted in the widespread interest in the fabrication and characterization of this class of materials. The nature of the TI class of materials resulting from its unique spin nature is gaining in importance when one realizes that TIs properties may entail generation of quasiparticles and electronic states which are not accessible in classic condensed-matter systems. Not surprisingly the topological insulators are considered as promising materials for multiple applications in next generation electronic or spintronic devices [8,9] as well as for applications in energy conversion such as thermoelectrics [10,11].

It is well known that defects, strain, and doping influence are important features of the TIs. Coulomb, magnetic, or disorder perturbations can modify the surface states [12,13,14]. Elemental doping and alloying in the TI materials allows for the control of the Fermi level via population with the n-type or p-type carriers which is also a crucial step towards the future junctions of topological insulators that associate two doped TI layers [15,16]. Among doped TIs, special interest is placed on magnetic dopants, mostly due to the possible interplay between topological magnetic orders, which may lead to the implementation of exotic quantum phenomena, such as the magnetoelectric effect [17] and quantum anomalous Hall effect (QAHE) [18,19], and later on the exploration of its potential device applications. Recent research is focused mostly on TI’s electronic structure and their thermoelectric properties and include structures containing transition metals dopants like V, Mn, Cr, Fe, and Cu [20,21,22,23,24,25], and rare earth dopants Gd, Ce, Y, Sm, Dy, and Eu [8,9,10]. Mn and Fe doping of the Bi_2_Te_3_ causes the opening of a small gap at the Dirac point in the surface Dirac cone [22]. Further, with the formation of ferromagnetic state only at the surface, a state which leads to opening the gap, has been confirmed for the Mn doped Bi_2_(Te,Se)_3_ as well as for the Cr-doped (Bi,Sb)_2_Te_3_ thin films [26]. Bi_2_Te_3_ doped with rare earth is discussed mostly from the point of view of modifying the thermoelectric properties [27,28,29,30,31,32,33]. Controlling the distribution of magnetic dopants within the TI’s matrix resulting from sample preparation procedures and chemical potentials of the constituent atoms is still challenging and requires in-depth and careful characterizations at the atomic level. This is especially the case when considering nanoscale systems, where already a controlled fabrication, quality control, as well as subsequent preservation of the completed structure or even device requires specific attention.

In our previous paper [34], we have studied the multilayered structures of Bi_2_Te_3_ and europium and shown that the interface between the layers is not stable and the reaction that took place at room temperature led to the decomposition of the originally layered structure and formation of new phases. This observation led us to focus on the electronic and crystallographic structures of a simplified structure where europium is doped in the Bi_2_Te_3_ thin film. Europium by itself is quite chemically active, and possesses interesting magnetic properties related to its valence state. It is worth noting that it is reasonable, at a first glance, to assume that europium atoms will partially substitute the bismuth atoms in the Bi_2_Te_3_ matrix as it happens for the Bi_2_Te_3_ doped with Gd [35,36]. Recently, the homogeneous incorporation of Eu^2+^ into the Bi sites of the Bi_2_Te_3_ lattice was observed [37]. Our point of interest was strongly aimed at the electronic structure of the obtained film, especially on the valence state of europium. Europium in alloys and compounds occurs in 2+ and 3+ valence states. Eu^3+^ is non-magnetic (J = 0) while the Eu^2+^, observed for pure Eu and europium in intermetallic alloys and compounds [38,39], has a large pure spin moment (J = 7/2). A mixed valence state has also been found in many intermetallic compounds. In the presented studies, the response of thin film of Bi_2_Te_3_ to the disorder introduced by Eu dopant is discussed. The studies were performed on thin films with an unprecedented combination of techniques such as photoemission spectroscopy, TOF-SIMS, RHEED, femtosecond spectroscopy, and LC-AFM.

## 2. Materials and Methods

### 2.1. Materials

The molecular beam epitaxy system was used for the growth of two thin films of Bi_2_Te_3_. The films were grown in accordance with the procedure we developed earlier [40] for the Bi_2_Te_3_ monocrystalline films. Freshly cleaved (110) 0.15–0.17 mm thick Muscovite mica substrates (Ted Pella, Inc., Mica grade V1) was used as a substrate for the Bi_2_Te_3_ films. In order to prevent the problem of the sample surface charging resulting from the used substrate, the mica surface was ground using silver paste. The mica was outgassed at 300 °C for 1.5 h under UVH conditions (5 × 10^−9^ mbar) and kept at 120 °C during the deposition of the Bi_2_Te_3_ films. High-purity Bi (99.999% Aldrich Chem. Co., Milwaukee, WI, USA), spectrographically standardized Te ingot (Johnson Matthey Chemicals, London, UK), and europium were evaporated by conventional effusion Knudsen cells (K-cell). The growth rate of each element, controlled by quartz microbalance, was customized to obtain assumed stoichiometry. The total growth rate of the films was about 0.2 QL min^−1^. In order to obtain a uniform distribution of deposited elements, the films were grown in the co-deposition mode. For the europium doped Bi_2_Te_3_ film, the growth rates of elements were adjusted as if Eu atoms were to substitute for bismuth in the Bi_2_Te_3_ crystal lattice. Assumed level of europium dopants was set at the level of about 2–3%. After the deposition process the films were annealed at 100 °C for 18 h. During the deposition process, the vacuum was of about 4 × 10^−9^ mbar for the undoped film and about 9 × 10^−9^ mbar for Bi_2_Te_3_ film doped with europium. Further measurements were performed on films cooled to room temperature. Additionally, pure, bulk europium was used as a reference sample for the photoelectron spectroscopy measurements.

### 2.2. Characterization Methods

In-situ structural characterization of the deposited film was realized with the use of reflection high energy electron diffraction (RHEED, STEIB Instrument, Langenbach, Germany) and X-Ray Photoelectron Spectroscopy. The RHEED diffraction patterns were obtained using an electron gun set at 15 kV, 1.4 mA along [100] direction after annealing and cooling down to room temperature. The electronic structure studies were conducted in-situ after the growth process using the XPS spectrometer (Physical Electronics PHI 5700, Chanhassen, MN, USA). For the XPS measurements, the monochromatic X-ray (1486.7 eV, Al Kα) radiation was used to generate photoelectron spectra; the survey spectra and high-resolution photoelectron spectra of Bi4f, Te3d, Eu3d, Eu4d, and valence band were collected at room temperature. The transmission electron microscopy (TEM) observations were performed using JEOL JEM-3010 high-resolution (HR-TEM) machine (Tokyo, Japan) working with 300 kV acceleration voltage and equipped with a Gatan 2k × 2k Orius™ 833 SC200D CCD camera (Gatan-Pleasanton, CA, USA). Atomic force microscopy (AFM) (Omicron VT AFM/STM, Taunusstein, Germany) was performed ex-situ under UHV condition at room temperature. AFM in tapping mode was used to study the layer growth process with high lateral resolution while the contact mode (LC-AFM) with conducting tip was applied to examine the character of the local conductivity at various polarization and bias voltage values up to 50 mV. Time of flight-secondary ion mass spectrometry (TOF–SIMS) measurements were carried out with the use of a TOF-SIMS.5 (ION-TOF GmbH, Munster, Germany) reflection-type spectrometer, equipped with bismuth liquid metal ion gun and cesium gun. 2D micro-and 3D nanostructure was examined using Bi^+^ ion beam (30 kV, 0.5 pA) for the analysis, and Cs^+^ (500 V, 100 nA) for depth profiling. Time-resolved optical measurements were performed using the femtosecond pump-probe technique, based on a 80 MHz repetition rate Ti:sapphire femtosecond laser (120 fs). The details of the set-up can be found in Ref. [41,42]. This permits to probe the impact on the Eu doping on the electron and phonon dynamics. For all ex-situ measurements, the film was measured on mica substrate without any additional preparation with the exception of TEM measurements. In that latter case, the film was partially mechanically exfoliated from the mica substrate and later placed on the copper mesh. The high-resolution images were obtained near the edge of the exfoliated film, where remaining mica support was thin or absent.

## 3. Results and Discussion

### 3.1. Crystalographic Structure

**Figure 1:**
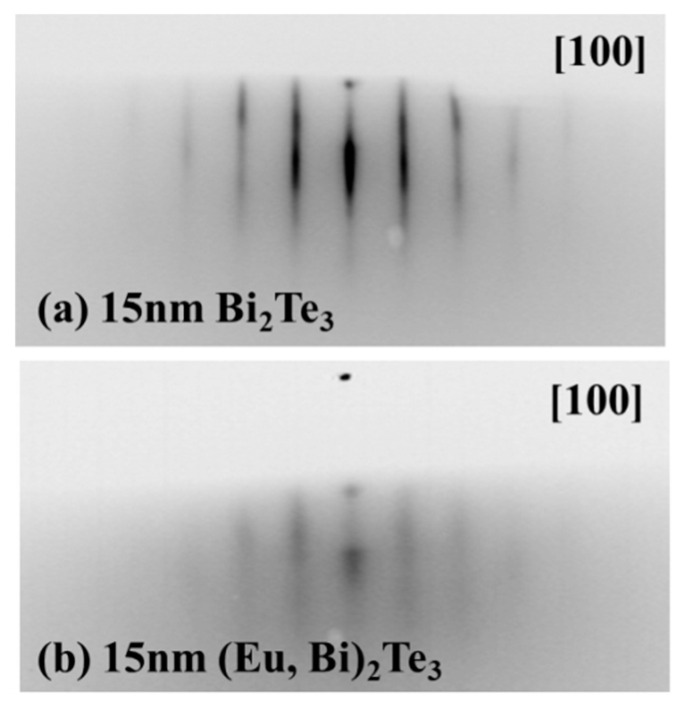
RHEED diffraction patterns of (**a**) 15 nm thick Bi_2_Te_3_ and (**b**) 15 nm thick (Bi, Eu)_2_Te_3_ film right after deposition process.

The crystallographic structure of obtained films was studied right after the deposition process with the use of a RHEED diffractometer. The results presented in Figure 1 indicate monocrystalline character of the undoped (Figure 1a) and Eu doped (Figure 1b) films. The in-plane lattice parameter calculated for the trigonal symmetry as in the Bi_2_Te_3_ structure (space group 166, $R\bar{3}m$) was 4.5 Å for the undoped film and 4.4 Å for the film doped with europium. Those values are in good agreement with the a = 4.38 Å parameter of the Bi_2_Te_3_ compound. The europium doping caused a slight expansion of the Bi_2_Te_3_ unit cell. Moreover, analyzing the RHEED images, a blurring of the diffraction pattern that can be observed for the Eu doped film is most likely related to weakly developed monocrystalline grains.

**Figure 2:**
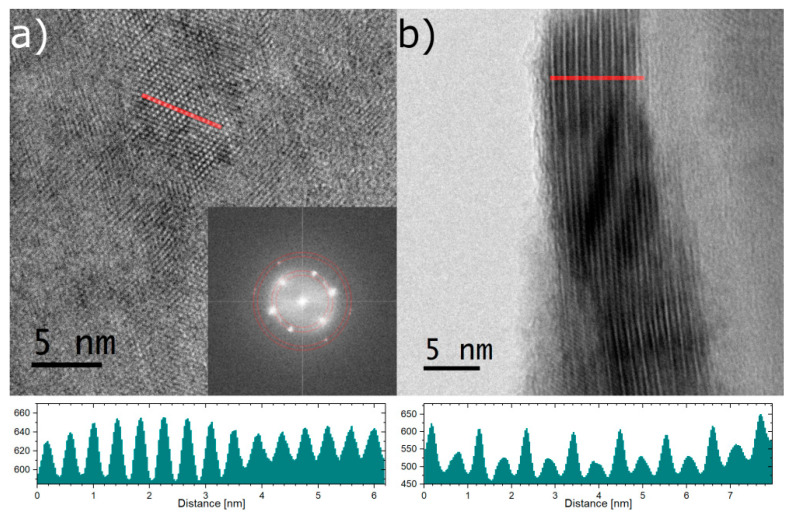
The figure presents transmission electron microscopy high-resolution images of the studied sample. (**a**) shows image perpendicular to the obtained layers. The crystalline character can be clearly visible. The inset shows Fourier Transform with marked by red circles interplanar distances corresponding to the Bi_2_Te_3_ structure. (**b**) shows area of the sample where the coating was bent showing the correct arrangement of the layers. Below line profiles of the regions marked by red lines are presented. One can easily notice the periodic structure with interplanar distances belonging to the Bi_2_Te_3_ structure.

High-resolution TEM images of the film surface aligned perpendicular to the electron beam as well as obtained diffraction patterns indicate the presence of monocrystalline ordering of the atoms in the film (Figure 2a). The grains have rather an irregular shape, their size varies between 8–25 nm, however, the grains are oriented towards each other at an angle of 60 degrees. Such an arrangement of the single crystalline grains is a favorable situation to obtain a RHEED pattern typical to a macroscopic single crystal as presented in Figure 1. This would also explain blurring of the RHEED pattern of Eu doped film. The trigonal symmetry is perfectly confirmed when looking at the atomic scale. The in-plane lattice parameter was of the same value as given for pure Bi_2_Te_3_ [43] and value obtained in our previous paper [40]. The high-resolution TEM image shown in Figure 2b represents an image obtained with the electron beam aligned parallel to the film surface. The visible stripes quite accurately show a layered structure of the film. The distance between those features is about 1 nm and is in good agreement with the thickness of the single quintuple layer [44,45].

### 3.2. Electronic Structure

The structural characterization was completed with the analysis of the electronic structure studied in terms of photoelectron spectroscopy. The studies were performed in-situ after the deposition process and compared to that of the undoped Bi_2_Te_3_ film and metallic Eu. Both Bi_2_Te_3_ films were obtained in the same system at the equivalent conditions. Both had a thickness of about 15 nm. Bulk Eu was prepared in the UHV conditions by scraping with a diamond file. No contamination by oxygen nor carbon was detected. The XPS core level spectra Bi 4f_5/2,_ Te 3d and Eu 4d were used to calculate the atomic composition which was Te:Bi:Eu = 55.3:41.3:3.4. The calculations were performed using Multipak v. 9.6 software, the Shirley method of background removal was applied. The ratio Te/(Bi + Eu) is slightly lower than expected but similar composition was found for high quality Bi_2_Te_3_ films [40,41].

**Figure 3:**
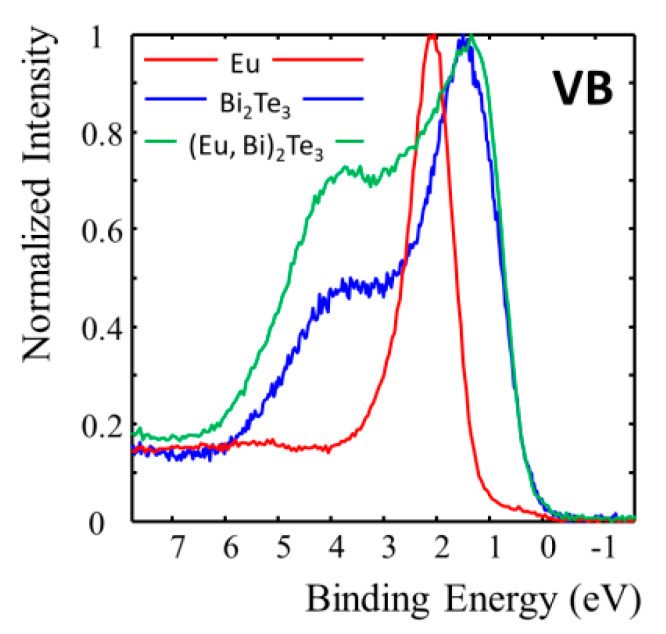
Valence band region for the pure europium (red line), 15 nm thick Bi_2_Te_3_ (blue line) and Eu doped Bi_2_Te_3_ (green line) film.

In the valence band region (see Figure 3), the difference between the undoped and Eu doped film is mostly pronounced in the binding energy range around 1 eV, close to the top of the valence band and in the region of about 3–4 eV. That difference may be related with the contribution from the Eu 4f states which have a high sensitivity factor for photoionization and despite the low Eu content give a relative increase of intensity in the region of about 1–4 eV. The Eu 4f states in the pure Eu are visible as a clear peak with a maximum at about 2.1 eV. It is worth mentioning that Denecke et al. reported the position of the Eu 4f peak at about 1.5 eV for the (Pb,Eu)Te bulk system [46]. The region close to the Fermi level, where the surface states plus possibly bottom of the conduction band are located, is hardly affected by Eu doping. This is in accordance with the measured electrical properties of the film and the transient reflectivity data described below. Eu remains in the divalent state, which can be clearly seen in the exchange split core d levels and by the lack of trivalent Eu 4f contribution in the region of binding energy 5–10 eV [47].

**Figure 4:**
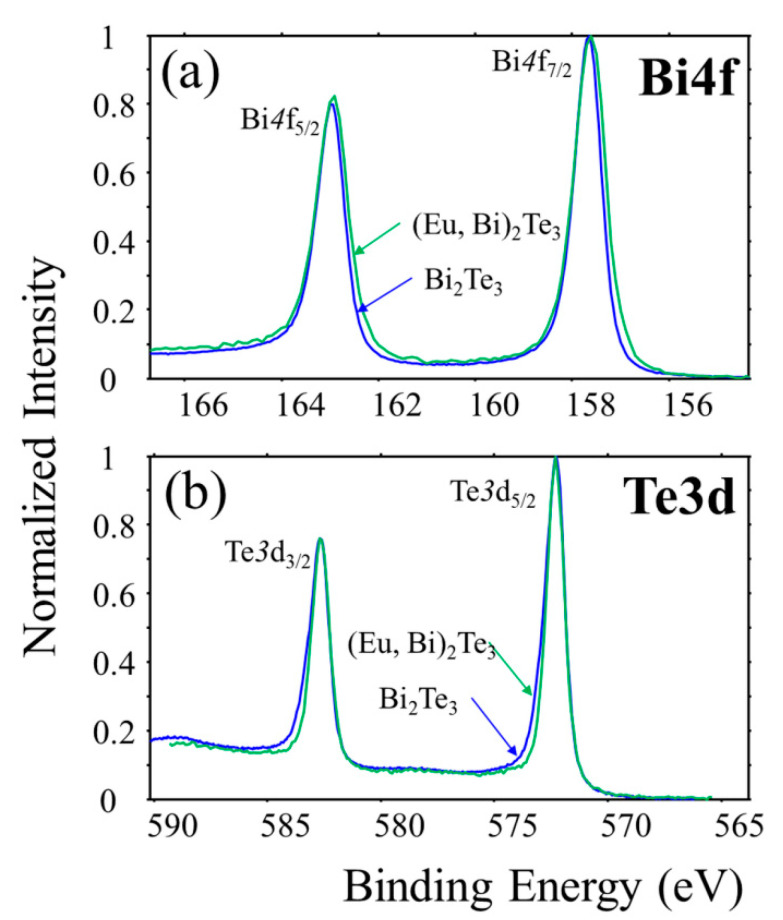
XPS spectra of (**a**) Bi 4f and (**b**) Te 3d levels for undoped and Eu doped Bi_2_Te_3_ films.

The chemical shifts of Te and Bi in undoped films show the expected behavior where the chemical shift of Bi is positive and that of Te is negative with respect to pure elements. The position of the Bi 4f and Te 3d photoemission levels is the same as for the undoped film. There is a slight difference in the line width as the Bi line is broader in the doped film (see Figure 4a) while the Te one is slightly narrower (Figure 4b). A slight broadening of the Bi lines can be related with a modified bonding when the nearest neighbor is Eu atom.

**Figure 5:**
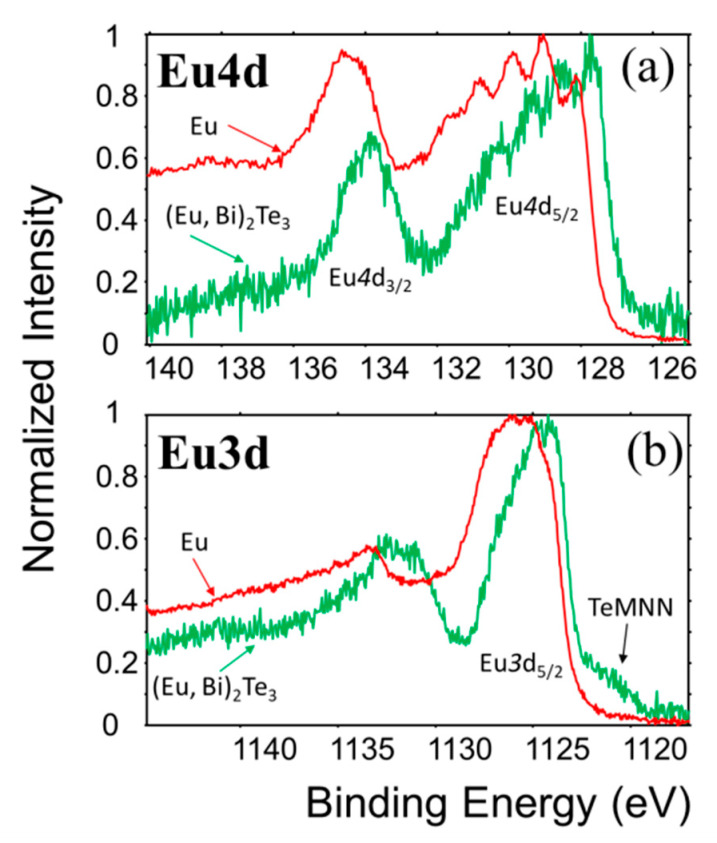
XPS spectra of Eu 3d (**a**) and Eu 4d (**b**) multiplets for pure europium and europium in (Eu, Bi)_2_Te_3_ film.

The very interesting point is the negative chemical shift of the Eu 4f level which is visible for Eu core levels. Figure 5 shows the comparison of the Eu 4d and 3d spectra obtained for the doped film with pure Eu. The shape of the core levels and well resolved exchange splitting confirms the divalent state indicating the presence of half occupied 4f shell. Assuming the stable ^7^S ground state for the Eu 4f shell, the negative shift may be related with the reconfiguration of the conduction electrons leading to the reduction of screening of the nuclear charge. This is surprising given the presence of Te ligands which have a much higher value of electronegativity. For the Eu 4d multiplet (see Figure 5a) the general structure is the same as for Eu metal except the intensity ratio of the first exchange split lines [48]. The splitting, i.e., distance between the first five well resolved lines within the main 4d_5/2_ peak is of about 0.85 eV what is very close to the value for Eu metal. The first peak which can be ascribed to the ^9^D_6_ term of the 4d^9^4f^7^ configuration has an Eu metal lower intensity than expected from the quantum multiplicity of states. The same effect was found for Gd and its metallic compounds, and remains unexplained up to date [49,50]. The intensity ratio of the 4d multiplet components (ratio of two lowest energy lines) observed in Eu doped Bi_2_Te_3_ film is thus exceptional. Similar structure was only found in early studies on bulk EuTe [51] and recent studies of europium incorporated in Bi_2_Te_3_ matrix [37]. That exceptional behavior can be related to the spin dependent relaxation rates of the photoexcited 4d states. The lowest spin dependent relaxation rate is expected for the state with the highest spin polarization and lowest binding energy within the mutiplet [49]. The lack of the effect in Eu doped Bi_2_Te_3_ may be related to the modified electronic structure close to the Fermi energy although the ground state of the Eu 4f level is ^7^S_0_ (divalent Eu as for Eu metal), leading to the well resolved exchange splitting and spin polarization of all core levels. 

### 3.3. TOF-SIMS Depth Profiles

After removing the (Eu, Bi)_2_Te_3_ film from the growth chamber, the film was transported under atmospheric conditions to the vacuum chambers of the mass spectrometer and atomic force microscope, the transport process took no more than a few minutes. The ex-situ TOF-SIMS characterization was focused on the analysis of the spatial distribution of layer components. It should be remembered that the TOF-SIMS technique, unlike the XPS, does not provide information on the atomic concentration of layer components, here the number of detected ions is related to the ionization probability and not to the real amount of particular element in the film. Moreover, the depth of analysis for mass spectrometry is about an order of magnitude smaller than for photoelectron spectroscopy.

**Figure 6:**
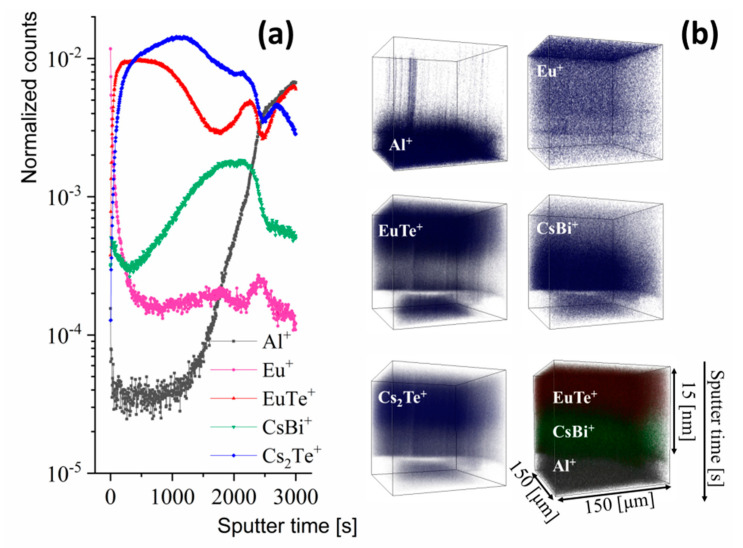
TOF-SIMS depth profiles of Eu doped Bi2Te3 films. Linear profile of film components (**a**), and 3D depth profiles (**b**) for selected secondary ions.

Figure 6a presents depth profiles indicating that the distribution of film components is not uniform as it supposed to be in a homogenously doped film. Europium ions exhibit a tendency to be uniformly distributed within the film, however in the upper part of the layer large amount of them was detected. A larger amount of europium at the surface of the film is probably related to surface segregation during the film growth or to the annealing process and, to a small extent, related to the oxidation of the film surface, an effect of migration towards the sample surface and further oxidation of the Eu. Interestingly, the presence and the distribution of the EuTe^+^ ionic species indicate that a relatively thick layer containing Eu and Te was formed in the top part of the film. As this layer also contains bismuth, one can relate that part of the film to the main Bi_2_Te_3_ phase with europium substituting bismuth. This is in agreement with the XPS data for the as grown sample. Regarding the analysis of the distribution of Te and Bi atoms, we used Cs_2_Te^+^ ionic species and BiTe^+^. The cesium forms that kind of ionic species with Te and Bi and allows to increase the signal derived from secondary ions of those elements [52]. The distribution of tellurium represented by the Cs_2_Te^+^ ionic species is not uniform. The linear depth profile and 3D distribution show that tellurium occurs in the entire film except for the sample surface, it can be also seen that a local maximum of Te atoms is present in the middle part of the film, however, it is not in the same place where the maximum for EuTe^+^ was observed. The bismuth in the analyzed film, represented by CsBi^+^ species, is partially accumulated in the area near the mica substrate. It seems that the formation of sub layer from which EuTe^+^ ions have been emitted leads to the displacement of some amount of the bismuth atoms towards the substrate. This result together with described above results of structural analysis confirms substituting Eu in the Bi_2_Te_3_ lattice. In our previous work [34] we already observed similar displacement of Bi atoms in the multi-layered system. Surface oxidation, typical for rare earth doped Bi_2_Te_3_ thin films [32], may, as in ex-situ measurements performed by us, enhance the segregation and substitution effect. Analysis of the Al^+^ signal, representing the mica substrate, indicate that the surface of the mica is not fully flat, the features (see Figure 6b, Al^+^) visible in the top part of the 3D depth profile suggest the presence of small island or crumbs on the surface of mica. That is also the reason why the interface between the mica and deposited film is not sharp as can be seen in Figure 6a for the Al^+^ linear profile.

### 3.4. Phonon-Electron Interactions

**Figure 7:**
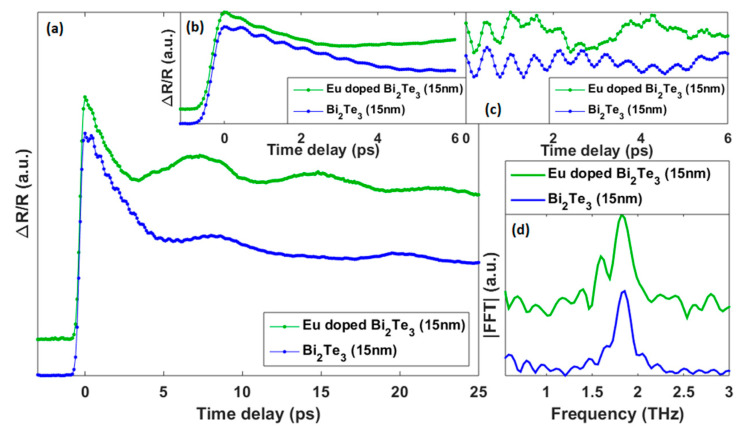
(**a**) Transient optical reflectivity measured for doped and undoped Bi_2_Te_3_ film. (**b**) Zoom on the first 6 ps time windows. (**c**) Coherent A1g optical phonon signal. (**d**) Fast Fourier Transform of the signal shown in (c).

**Figure 8:**
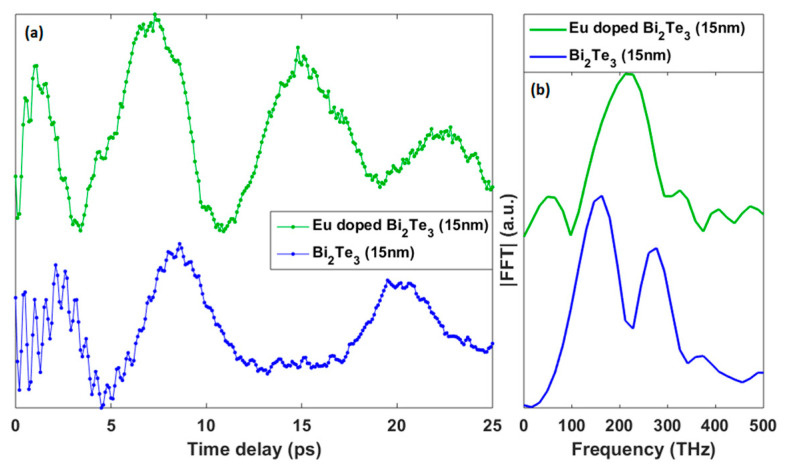
(**a**) Coherent acoustic phonon signals extracted from the transient optical reflectivity signals (see Figure 7a) for undoped and Eu doped Bi_2_Te_3_ film. (**b**) Corresponding coherent acoustic phonon spectra obtained by a fast Fourier transform (FFT).

To complete the electronic and structural characterization, time-resolved optical measurements have been conducted to evaluate the electron–phonon collision time and to assess the impact of such chemical and structural disorder onto the phonon dynamics. The Eu doped Bi_2_Te_3_ film was investigated after a few weeks after the deposition process. A stable oxidation layer appears, as already evidenced earlier [41,42]. This oxide layer thickness was estimated to be around 2 nm [41,42]. We have then measured the transient optical reflectivity signal of two films with and without Eu doping. The signals are shown in Figure 7. The optical penetration of both the pump and probe light is similar to the film thickness, so we excite and probe the entire thickness. Moreover, the light impinges on the surface with a diameter of around 5 micrometers. So, the information that such technique obtains is an average response over that photoexcited volume. We do not have in plane resolution, however, since we are able to record time of flight of phonons (acoustic) we are sensitive to in-depth inhomogeneities as we will discuss in the following [53,54,55]. The transient optical reflectivity (Figure 7) exhibits a fast and sharp variation just after the femtosecond excitation due to the ultrafast excitation of the electronic cloud. Then, the signal decays in time due to the relaxation of hot carriers and to heat diffusion process. Some oscillating components superimpose on the electronic response (Figure 7a) and can be evidenced more clearly once the baseline is removed (Figure 7c and Figure 8a). The oscillatory components come from a photo-induced coherent optical phonon (THz range as seen in Figure 7c,d) and a coherent acoustic phonon (sub-THz range as seen in Figure 8a,b). The assignment of these oscillations has been previously established for undoped Bi_2_Te_3_ [41,42]. The phonons signal is extracted after removing the baseline. We have recorded the signal with a broad time window (0–30 ps) with low time resolution sufficient to record the acoustic phonon signals (Figure 8). In contrast, for evidencing more clearly the high frequency phonons, we have increased the time resolution of the record (refined sampling) but we have limited the record to the first 8ps (Figure 7b,c). 

As observed in Figure 7a,b within the first 5ps, the signal decays rapidly, which is due to the hot carriers’ relaxation. A time of typically 2 ps is found consistent with previous reports [41,42]. We note that such electron-phonon relaxation time depends on the electronic structure close to the Fermi level since the photoexcited carriers are promoted towards empty states with quanta of the pump excitation of 1.5 eV and relaxes towards the initial states afterward. In Figure 3, we have shown that the valence band structure down to 2 eV below the Fermi level was not too different for the doped and undoped layers. Consequently, we are not surprised to measure equivalent cooling time for Eu-doped and undoped samples. However, the doping seems to have a drastic influence on the phonon dynamics as discussed hereafter.

The optical mode has a frequency close to 2THz (Raman active A_1g_ mode seen in Figure 7d) in agreement with previous investigations [41,42]. The fact that we detect the same A_1g_ frequency as the one measured in undoped Bi_2_Te_3_ materials, is an additional confirmation that the right structure has been grown consistently with a conclusion drawn before. We note that such optical phonon frequency is governed by the local interatomic potential and, for this reason, is a good local probe of a structure. However, we can see a huge difference in the lifetime of these coherent optical phonons. While for undoped sample the coherent optical phonon is visible beyond the time window of observation (8ps), its lifetime drastically decreases for the doped sample. This A_1g_ optical phonons have a very low group velocity. As a matter of fact, the observed limited lifetime cannot be attributed to the scattering at grain boundaries or at any defect present in the structure since this A_1g_ phonons do not propagate (contrary to acoustic phonon as discussed later on). Rather, the lifetime is likely associated to local fluctuations of chemical compositions that can modify the interatomic potential and hence modify the intrinsic A_1g_ frequency (slight change). Consequently, the total signal arises from contributions of slightly different A_1g_ frequency coming from unaffected and affected (due to the doping) sites of Bi_2_Te_3_ (the A_1g_ mode is associated to longitudinal displacement within the QL). At this level, we cannot detect the slight difference in the A_1g_ frequency in the time domain, but such different cosines like signals interfere thus reducing the coherence of the signal in the time domain, i.e., reducing the lifetime. This effect is well known, referred to as inhomogeneous broadening, and has been already observed when alloying some semiconductors or where slight disorder exists in a structure [56]. So, we can conclude that the doping creates such inhomogeneous broadening due to the perturbation of the local interatomic potential due to the insertion of Eu within the Bi_2_Te_3_ lattice. Since we have shown previously that Eu mainly substitute to Bi, such fluctuations of the interatomic potential can be reasonably attributed to that substitution. The impact of the doping on the elastic properties and scattering of acoustic phonon is another property we reveal. The acoustic phonons signals are very different (Figure 8). These acoustic modes are acoustic eigenmodes as shown in previous reports [42,57]. Said differently, these acoustic frequencies correspond to the light-induced mechanical resonances of the entire film. The mechanical resonance of a film having a thickness H and a longitudinal sound velocity V, is given by f_n_ = nV/2H. There is an important difference to be underlined. For the non-doped sample, we can evidence two eigenmodes (around f_n = 1_ = 80 GHz and f_n = 2_ = 160 GHz), as already observed previously [41]. We have repeated the experiment on the same sample studied in 2015 to show the stability of the sample and to confirm the acoustic phonon frequency did not change [41]. In contrast, for the Eu-doped sample, only one mode is clearly revealed with a frequency shifted upward (around 120 GHz compared to 80 GHz). Both films have a nominal thickness of 15 nm after the MBE growth (the thickness of undoped sample was measured by X-ray Reflectivity see [41]). We obtain a sound velocity of 2400 m/s for the undoped Bi_2_Te_3_ while we arrive to a value around 3600 m/s for the doped sample. This difference is considerable. We have noticed that the A1g frequencies (Figure 7) are pretty identical, indicating that within the QL the interatomic potential might not change too much upon doping.

The reasons for such a large variation of the acoustic phonon velocity might either be due to the reduction of the Bi_2_Te_3_ layer thickness and/or a partial segregation of the dopant or other chemical species relocated between QLs. Such reduction of thickness H, immediately induces an increase of the eigenfrequency f_n_. This scenario could be consistent with TOF-SIMS results where some layers with various chemical composition appear to exist. The other possibility, if we rule out the change of H parameter, could come from a partial segregation of the dopant or other chemical species between QLs. In that case, the sound propagation which is governed by the interatomic interaction between QLs might be affected. 

### 3.5. Local Conductivity

The closing investigation allowed exploring the surface topography and the local conductivity properties on the surface of the Eu doped Bi_2_Te_3_ film for which we also reveal some interesting spatially inhomogeneous physical properties. The AFM measurements were performed on the external AFM system, and thus the sample was briefly introduced to the atmosphere. It was shown [39], that the short introduction to the ambient conditions had a limited impact on the surface electrical properties. The investigations showed a relatively flat surface with a number of larger islands randomly present on the surface. The size of those islands is between 100 and 200 nm, however they also can be found packed in close formations of 300 nm size on average. The maximal island height is close to 20 nm. The average RMS value in the regions without the islands was very low and close to 0.8(1) Å, otherwise (regions with islands) the RMS is close to 3.0–5.0 nm. We did not observe clear triangular-shaped islands (contrary to undoped films [40]), however an island-like growth mechanism still occurs.

**Figure 9:**
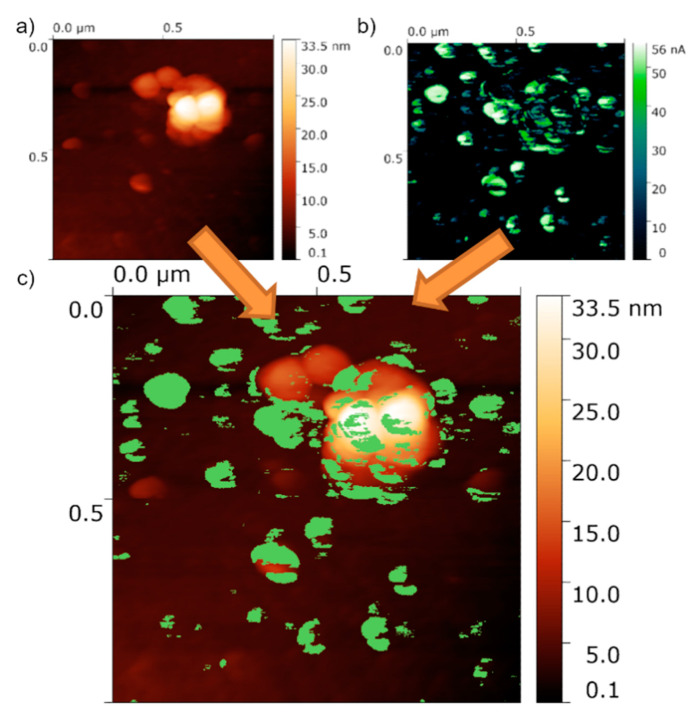
Typical surface of the Eu-doped Bi_2_Te_3_ thin film measured by the LC-AFM: (**a**) the topography, (**b**) local current map, (**c**) combination of topography and the local current.

Further, we have investigated the local current maps with the use of LC-AFM–where the conducting AFM tip is in the contact with the surface under a small voltage (several mV). This allows for the investigation of the local conductivity of the surface along with its topography. The typical results for the LC-AFM are shown in Figure 9. The map shows that the surface conductivity is inhomogeneous and is correlated with the topography–Figure 9c). The interpretation of such conductivity can be drawn with a comparison to the undoped sample surface (see local conductivity maps in [40]). First, the regions between the islands are conducting poorly, which is the opposite to the expectation of a topological insulator surface (pure Bi_2_Te_3_ thin film). From the TOF-SIMS technique, one can notice that the Eu content on (or close to) the surface is high. Thus, poor conductivity in such regions can originate from that fact. Eu is very susceptible to oxidation and a layer of EuO or Eu_2_O_3_ is formed. The EuO is semiconducting [58], while the Eu_2_O_3_ is insulating giving again very low currents on a current map. Moreover, in the compounds with the divalent Eu, like EuO or EuTe relatively strong magnetic interactions can appear leading to the destruction of the topologically protected states and some regions on the surface become semiconducting. Hence, the relatively low voltage used in our experiment (several mV) results in very low currents on a current map. At the same time, the regions close to the large islands are conducting very well, giving low resistance in the range of 80 kΩ. The resistivity connected to the experimental setup in our LC-AFM can be estimated to 60 kΩ (which depends strongly on the tip-sample contact area). Thus, we estimate that the results of below 100 kΩ are very close to the lowest resistance that can be detected, thus can be interpreted as very large conductivity. In addition, the I–V curves show metallic conductivity, leading to an assumption that those regions conduct with a very high conductivity, which in turn is a hallmark of the topological insulator state. In addition, one can notice a number of small islands conducting poorly, especially those with a relatively low height. One may suppose that the presence of Eu rich phases can be responsible for that behavior. In summary, we assume that the large islands shown in the topography are composed of a main Bi_2_Te_3_ phase with a small Eu doping level, while the rest of the surface is covered by Eu containing phases, most likely oxides.

## 4. Conclusions

The impact of Eu doping on the structure and properties of Bi_2_Te_3_ was investigated. Our studies indicated that 15-nm thick film of Bi_2_Te_3_ doped with europium basically preserves the crystallographic structure of the undoped compound. The electron diffraction and TEM images, the measurement of the characteristic frequency of the A1g Raman active mode of Bi_2_Te_3,_ and the density of states probed by UPS-XPS confirm the average structure is successfully obtained. The in-situ XPS results indicate the divalent state of Eu, which means the presence of magnetic atoms within the lattice. Interestingly, the negative chemical shift of Eu core levels has been found and an exceptional structure of the exchange split Eu core levels has been observed. The magnetic interactions related to the magnetic atoms do not seem to influence the presence of surface states, as confirmed by a very high surface conductivity. However, some inhomogeneities have been revealed on the surface by AFM and LC-AFM, as well as in the body of the film thanks to TOF-SIMS and through the analysis of the coherent phonon dynamics. Even if the average structure is preserved, these inhomogeneities give rise to a complex landscape of surface conductivity and might affect some elastic properties of the layer, maybe due to some interstitial dopant between quintuple layers. The surface oxidation locally reduces the conductivity, which is probably connected to the presence of Eu oxides. Further optimization of the doped film growth process is necessary to obtain detailed knowledge concerning the effect of doping necessary to develop new classes of devices.
